# “Infectious uveitis: a comprehensive systematic review of emerging trends and molecular pathogenesis using network analysis”

**DOI:** 10.1186/s12348-024-00444-8

**Published:** 2024-11-20

**Authors:** Muhammad Arif Asghar, Shixin Tang, Li Ping Wong, Peizeng Yang, Qinjian Zhao

**Affiliations:** 1 College of Pharmacy, Chongqing Medical University, Chongqing, PR China; 2 College of Public Health, Chongqing Medical University, Chongqing, PR China; 3 Department of Social and Preventive Medicine, Faculty of Medicine, University of Malaya, Kuala Lumpur, 50603 Malaysia; 4 Chongqing Key Lab of Ophthalmology, Chongqing Branch of National Clinical Research Center for Ocular Diseases, The First Affiliated Hospital of Chongqing Medical University, Chongqing Eye Institute, Chongqing, China

**Keywords:** Infectious uveitis, Non-infectious uveitis, Research trend, Global prevalence, Pathogenesis, Protein-protein interaction network, Differential diagnosis and treatment

## Abstract

**Background:**

Infectious uveitis is a significant cause of visual impairment worldwide, caused by diverse pathogens such as viruses, bacteria, fungi, and parasites. Understanding its prevalence, etiology, pathogenesis, molecular mechanism, and clinical manifestations is essential for effective diagnosis and management.

**Methods:**

A systematic literature search was conducted using PubMed, Google Scholar, Web of Science, Scopus, and Embase, focusing on studies published in the last fifteen years from 2009 to 2023. Keywords included “uveitis,” “infectious uveitis,” “viral uveitis,” and others. Rigorous inclusion and exclusion criteria were applied, and data were synthesized thematically. Gene symbols related to infectious uveitis were analyzed using protein-protein interaction (PPI) networks and pathway analyses to uncover molecular mechanisms associated with infectious uveitis.

**Results:**

The search from different databases yielded 97 eligible studies. The review identified a significant rise in publications on infectious uveitis, particularly viral uveitis, over the past fifteen years. Infectious uveitis prevalence varies geographically, with high rates in developing regions due to systemic infections and limited diagnostic resources. Etiologies include viruses (39%), bacteria (17%), and other pathogens, substantially impacting adults aged 20–50 years. Pathogenesis involves complex interactions between infectious agents and the ocular immune response, with key roles for cytokines and chemokines. The PPI network highlighted IFNG, IL6, TNF, and CD4 as central nodes. Enriched pathways included cytokine-cytokine receptor interaction and JAK-STAT signaling. Clinical manifestations range from anterior to posterior uveitis, with systemic symptoms often accompanying ocular signs. Diagnostic strategies encompass clinical evaluation, laboratory tests, and imaging, while management involves targeted antimicrobial therapy and anti-inflammatory agents.

**Conclusion:**

This review underscores the complexity of infectious uveitis, driven by diverse pathogens and influenced by various geographical and systemic factors. Molecular insights from PPI networks and pathway analyses provide a deeper understanding of its pathogenesis. Effective management requires comprehensive diagnostic approaches and targeted therapeutic strategies.

## Introduction

Uveitis, also known as intraocular inflammation, is a complex inflammatory condition that may affect the significant parts of the eye, including the ciliary body, iris, retina, vitreous, or choroid. It comprises over 25 disorders associated with the characteristic signs of intraocular inflammation [1]. Despite its classification as an orphan disease, uveitis is the fourth leading cause of blindness among the working-age population, highlighting its substantial impact on public health [2]. Unfortunately, its social and economic impact remains unreported in existing literature. Globally, uveitis accounts for approximately 10% of blindness cases in the Western world among working-age individuals [3, 4]. Although epidemiology and types of uveitis exhibit considerable variation, particularly in the Asian region, precise incidence rates remain to be determined in many parts of the world [5, 6].

Uveitis is classified anatomically based on the specific structures involved; it may manifest as anterior, intermediate, posterior, or panuveitis, each with distinct clinical features and treatment strategies [7]. The onset of uveitis can vary, ranging from abrupt to insidious, which poses significant challenges in diagnosis and management. Uveitis can be caused by various factors including infectious agents, immune-mediated, trauma, toxins, masquerade syndromes, and post-surgical complications [8].

Infectious uveitis arises from diverse pathogens, including bacteria, viruses, fungi, and parasites, each with its unique pathogenic mechanisms and clinical presentations. These infectious agents can access to the eye through various routes, such as hematogenous spread, direct inoculation, or reactivation of latent infections [9, 10]. Although non-infectious etiologies such as immune-mediated, systemic inflammatory conditions, and idiopathic factors contribute to a significant proportion of uveitis cases, infectious agents also play a crucial role, particularly in specific geographic regions and patient populations [11]. Understanding the epidemiology, clinical manifestations, molecular mechanisms and treatment principles of infectious uveitis is essential for ophthalmologists and healthcare providers to effectively manage this sight-threatening condition and preserve visual function in affected individuals. This review aims to provide a comprehensive overview of infectious uveitis, covering its recent research trend, prevalence, etiologies, pathogenesis, molecular mechanisms using the protein-protein interaction network analysis, clinical features, diagnostic approaches, and management strategies, focusing on recent advancements and emerging trends in the field. The novelty of this article lies in its synthesis of distinct sources, offering a cohesive narrative that not only highlights the diverse etiologies of infectious uveitis but also examines the complex molecular mechanisms underlying its pathogenesis. This review article on infectious uveitis could play a critical role in creating existing knowledge, highlighting recent advancements, and identifying gaps in understanding. It would be an invaluable resource for clinicians and researchers, facilitating evidence-based decision-making, fostering collaborations, and ultimately improving patient care and outcomes in uveitis management.

## Methodology

A systematic literature search was conducted utilizing several scientific databases, including PubMed, Google Scholar, Web of Science, Scopus, and Embase, to identify relevant studies published within the last fifteen years (2009–2023). The search was carried out between February 2024 and May 2024.

A comprehensive set of specific keywords was employed to refine the search, encompassing terms such as “uveitis,” “infectious uveitis,” “prevalence of infectious uveitis,” “etiologies of infectious uveitis,” “differential diagnosis of infectious uveitis,” “clinical patterns and treatments of infectious uveitis,” “viral uveitis,” “vector-borne diseases,” “acute retinal necrosis,” “dengue posterior uveitis,” “West Nile Virus chorioretinitis,” “Rift Valley retinitis,” “chikungunya posterior uveitis,” “Epstein Barr viral uveitis,” “Syphilitic uveitis,” “Ebola posterior uveitis,” “Zika posterior uveitis,” “Non-viral infectious uveitis,” and “polymerase chain reaction.” Initially, our search strategy focused on keywords primarily related to viral uveitis. However, we recognize the importance of including other common causes of infectious uveitis, such as ocular tuberculosis and ocular toxoplasmosis. Therefore, the search terms were expanded to include “ocular tuberculosis,” “ocular toxoplasmosis,” “bacterial uveitis,” “fungal uveitis,” and “parasitic uveitis” to ensure a more comprehensive review of all relevant infectious agents associated with uveitis. To enhance the completeness of the search and reduce bias, additional keywords related to non-infectious uveitis were also included, such as “non-infectious uveitis,” “immune-mediated uveitis,” “drug-induced uveitis,” “isolated ocular uveitis,” and “autoimmune uveitis,” allowing for a comparative analysis between infectious and non-infectious causes of uveitis. The expanded search strategy aimed to cover the full spectrum of uveitis types and minimize the risk of omitting relevant studies.

**Fig. 1:**
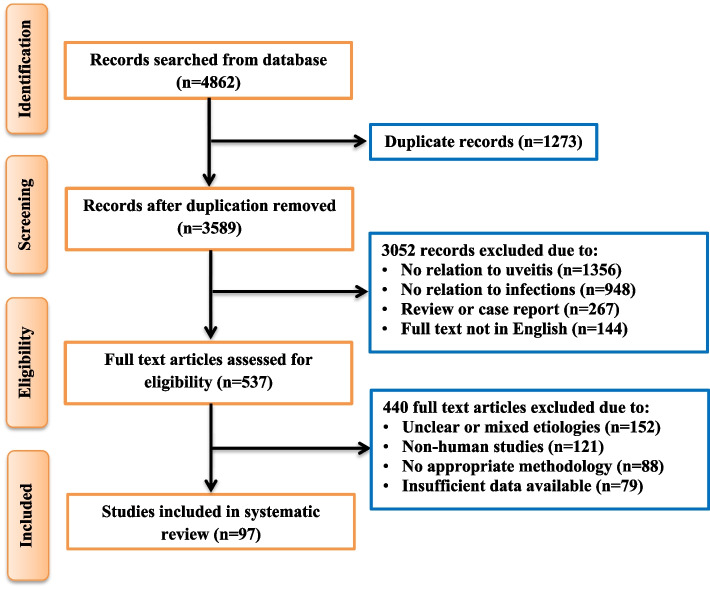
Flow and selection of preclinical studies (*n* = number of publications)

 The selection of research and review articles for inclusion in this review was based on a rigorous screening process. Initially, titles and abstracts were assessed for relevance to infectious uveitis. Articles were included according to the following criteria: (1) studies published in English between 2009 and 2023; (2) articles that provided substantial insights into the prevalence, etiology, pathogenesis, clinical features, diagnostic approaches, and management strategies for infectious uveitis; (3) both original research articles and systematic reviews were included to provide a broad perspective; (4) studies with sufficient methodological rigor, such as randomized controlled trials, cohort studies, case-control studies, and systematic reviews, were prioritized, and (5) any studies that used molecular or protein-protein interaction (PPI) network analysis and pathway analyses related to infectious uveitis. However, studies were excluded if they met any of the following criteria: (1) Articles with non-English language; (2) case reports, editorials, conference abstracts, and articles with insufficient sample sizes or unclear methodology; (3) non-humans studies or unclear etiologies; and (4) articles published before 2009 to maintain relevance with the latest research and developments in the field. A total of 97 articles met eligibility criteria according to the PRISM guidelines [12], while the complete screening and selection process of all relevant studies are presented in Fig. 1.

### Analysis of molecular mechanisms of infectious uveitis

The primary gene symbols underlying infectious uveitis were sourced from four databases: DisGeNET (https://www.disgenet.org/), GeneCards (https://www.genecards.org/), OMIM (https://omim.org/) and TTD (https://db.idrblab.net/ttd/). The selected intersecting genes were then uploaded into STRING 11.0 (https://string-db.org/cgi/input.pl/) to generate the protein-protein interaction (PPI) network for infectious uveitis. A high confidence score threshold (≥ 0.7) was applied to ensure the reliability of the interactions. The resultant network was visualized using Cytoscape software. However, the key topological parameters such as degree centrality, betweenness centrality, and closeness centrality were calculated to identify hub proteins within the network. The PPI network was visualized with nodes sized proportionally to their degree of connectivity and colored to reflect their centrality. Edges represented the interactions, with thickness indicating the interaction strength [13, 14].

### GO and KEGG pathway analysis

GO enrichment and KEGG pathway analysis were conducted using the DAVID Bioinformatics Resources 6.8 database (https://david.ncifcrf.gov/) to explore potential pathways associated with infectious uveitis. The results were visualized as bar plots showing fold enrichment and -log10(FDR) values, indicating statistical significance. FDR correction was applied to adjust for multiple testing, with FDR < 0.05 considered statistically significant [15, 16].

### Statistical analysis

False discovery rate (FDR) correction was applied to adjust for multiple testing in GO and KEGG enrichment analyses. A threshold of FDR < 0.05 was considered statistically significant. Fold enrichment was calculated as the ratio of observed to expected counts of proteins associated with a given term or pathway, providing insight into the degree of over-representation.

## Results and discussion

### Trends in research publications on infectious uveitis

**Fig. 2:**
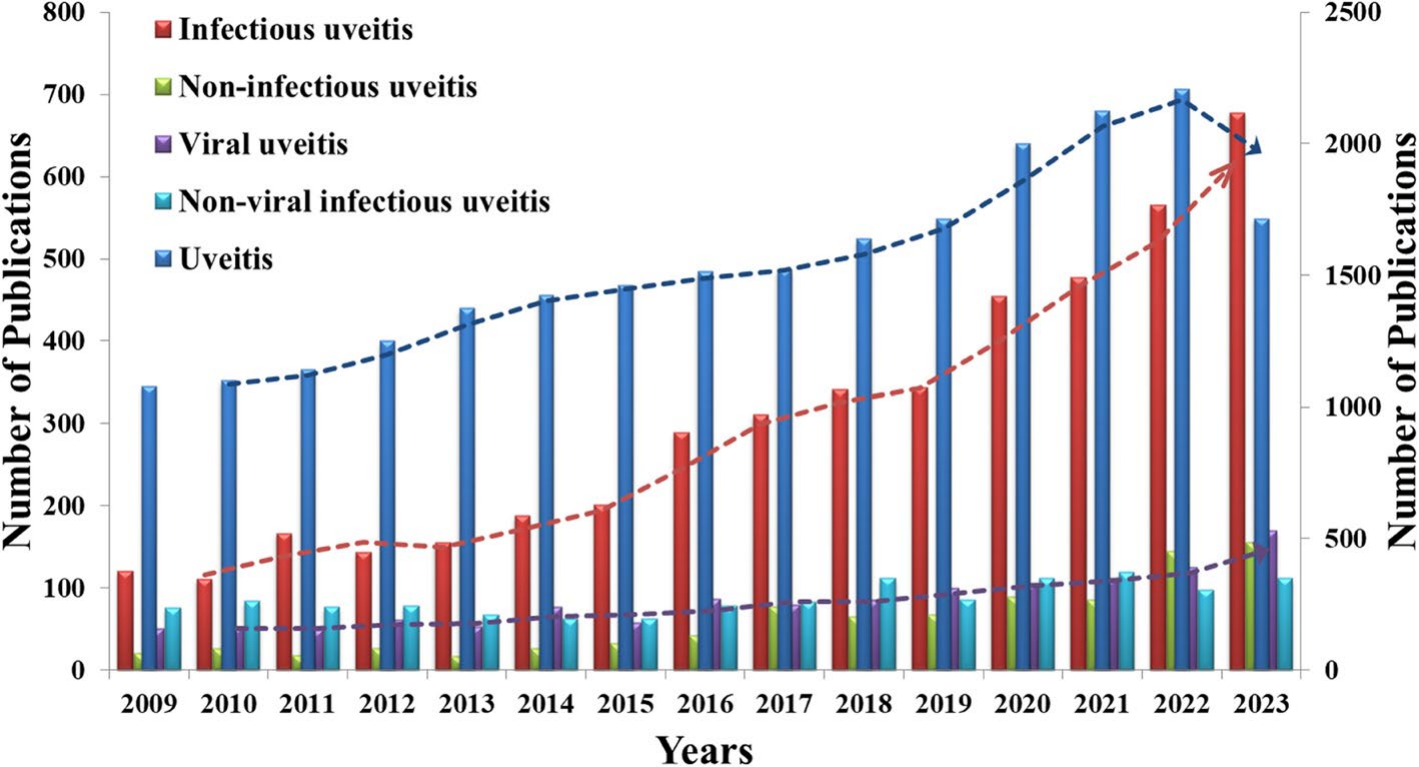
Publications trends of last 15 years (from 2009 to 2023) from PubMed on uveitis related research subjects

 The analysis of research publication trends over the last fifteen years, spanning from 2009 to 2023 in the PubMed database, reveals a significant rising pattern in the field of uveitis research, as showed in Fig. 2. The data presented in the figure describe the publication numbers across five distinct categories: uveitis, infectious uveitis, non-infectious uveitis, viral uveitis, and non-viral uveitis. Upon close examination, there was a notable surge in research publications on infectious uveitis. This trend indicates a growing interest in explaining the etiologies of infectious uveitis, specifically those related to viral pathogens. Compared to non-infectious uveitis, the emphasis on infections associated with uveitis highlights the evolving landscape of uveitis-related research. Infectious uveitis comprises a variety of manifestations, including both local and systemic infections, and the increasing trend in publication numbers within this specific category suggests a growing recognition of the pathogenesis of infectious-associated uveitis. The viral-associated uveitis emerges as a critical point for research, with a significant increase in publication numbers in recent years. Several notable factors could contribute to this increasing trend, such as increased awareness of different etiologies associated with infectious uveitis, advancements in diagnostic approaches, and the significant impact of infections globally in the last few years. However, non-infectious associated uveitis remains an important research area, as evidenced by several publications over the past fifteen years.

### Prevalence of infectious uveitis

**Fig. 3:**
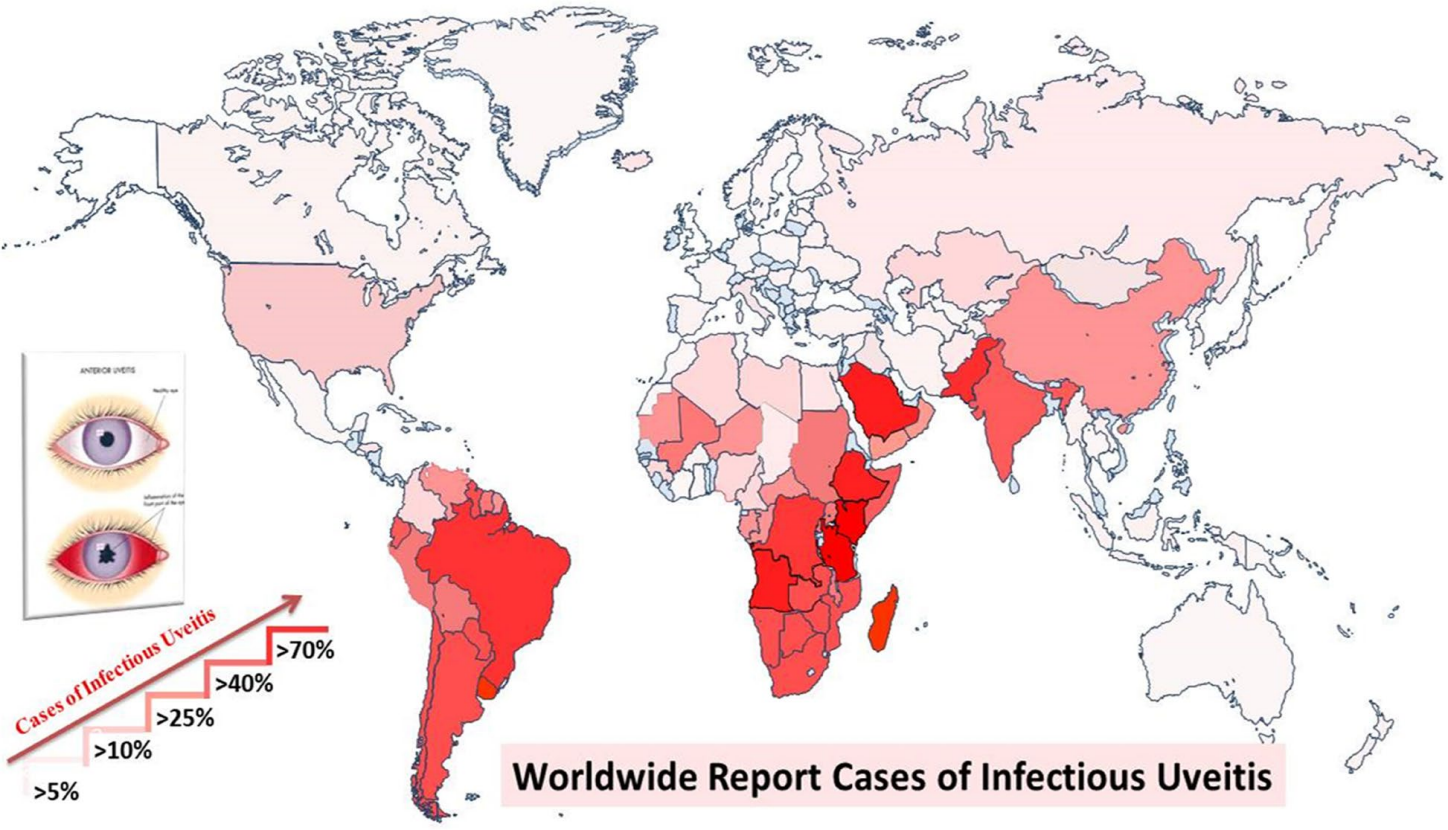
A global map representing the prevalence of infectious uveitis, with varying shades of red indicating different levels of prevalence. Areas with a lighter shade of red represent lower prevalence, while those with a darker shade of red indicate higher prevalence. This color gradient serves to visually illustrate the distribution and intensity of infectious uveitis across different regions worldwide (Prevalence rates may not be fully representative and lacked exact values due to differences in geographic regions, limited informations and diagnostic criteria)

 The prevalence of infectious uveitis exhibits significant variations influenced by various factors such as geographic location, environmental conditions, socioeconomic status, genetic predispositions, and histopathological characteristics. Notably, infectious uveitis accounts for up to 25% of cases of visual impairment in developing countries [17]. The estimated global prevalence rate of infectious uveitis gathered from previous studies is illustrated in Fig. 3. The global map revealing the prevalence of infectious uveitis highlights significant cases in specific regions, notably North and South America, Africa, and certain Asian countries. In South America, particularly in areas characterized by dense urban populations and inadequate healthcare access, the prevalence of infectious uveitis is markedly increased. For instance, Brazil exhibits a high prevalence rate of 81%, highlighting the considerable burden of this ocular disorder [9, 18, 19]. Across Africa, countries such as Ethiopia (77%), Tanzania (73%), South Africa (66%), and Angola (63%) exhibit a concerning prevalence of infectious uveitis, reflecting various socioeconomic and healthcare challenges prevalent in these regions [20–22]. Similarly, Asian countries like Pakistan (49%), India (37%), and China (21%), demonstrate significant burdens of infectious uveitis, attributed to factors such as population density, inadequate sanitation infrastructure, and limited healthcare resources [23–28]. Viral infections associated with uveitis are commonly found in the African and Asian regions and are associated with environmental and genetic factors, climate changes, and systemic non-infectious conditions [21, 29, 30]. The high prevalence in these areas highlights the urgent need for targeted interventions, including enhanced public health initiatives, improved access to medical care, and comprehensive strategies. The availability of modern treatment to manage infections and their related complications in developed countries has also altered the epidemiology of uveitis [31]. Due to inconsistencies in available data, the prevalence of infectious uveitis remains uncertain and should be interpreted with caution.

**Fig. 4:**
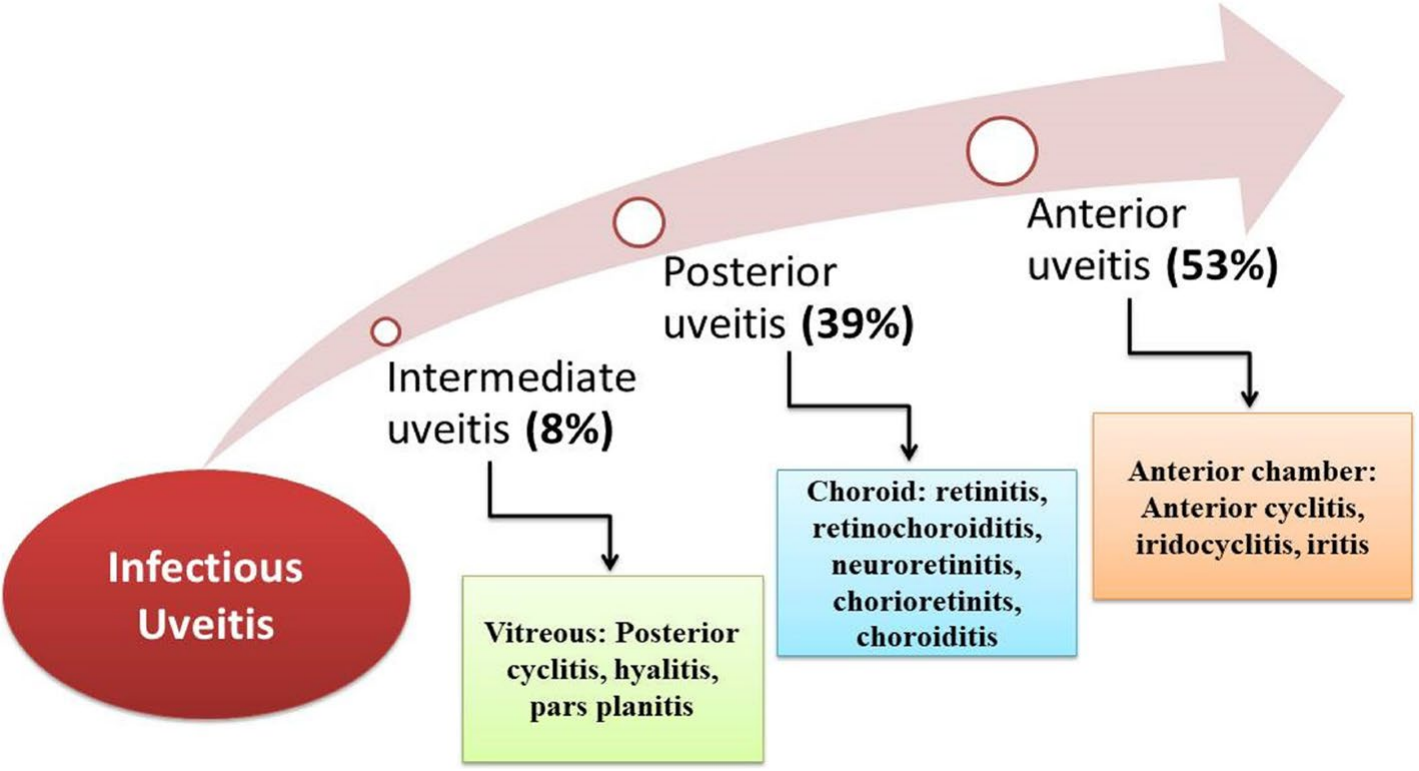
Estimated global prevalence infectious uveitis according to the ocular anatomical regions (Values are based on available studies, but variability exists due to differing research designs and populations)

 However, infectious uveitis exhibits distinct patterns concerning prevalence, affected anatomical regions, age distribution, and gender susceptibility. The data indicates that acute, non-granulomatous anterior cyclitis and iritis in the anterior region are the most prevalent forms of uveitis, i.e. 53%, as shown in Fig. 4 [31]. Subsequent types include retinitis, retinochoroiditis, neuroretinitis, choroiditis, and chorioretinitis linked with posterior uveitis (39%). Notably, posterior uveitis primarily stems from toxoplasmosis, cytomegalic virus (CMV) retinitis, and dengue maculopathy [32]. In addition, intermediate uveitis exhibits a lower prevalence (8%) compared to anterior and posterior uveitis but still contributes to the spectrum of infectious uveitis [20].

Regarding age distribution, adults aged 20–50 years are notably susceptible, accounting for 60–80% of cases within this age range, with an average onset age of around 40 years. Infectious uveitis is relatively rare in individuals under 10 years old and those over 70 [33, 34]. Gender differences in incidence rates are minimal, though a slightly higher prevalence is observed in females [23, 35]. Understanding these trends is vital for healthcare professionals in diagnosing, treating, and managing infectious uveitis.

### Etiology of infectious uveitis

**Fig. 5:**
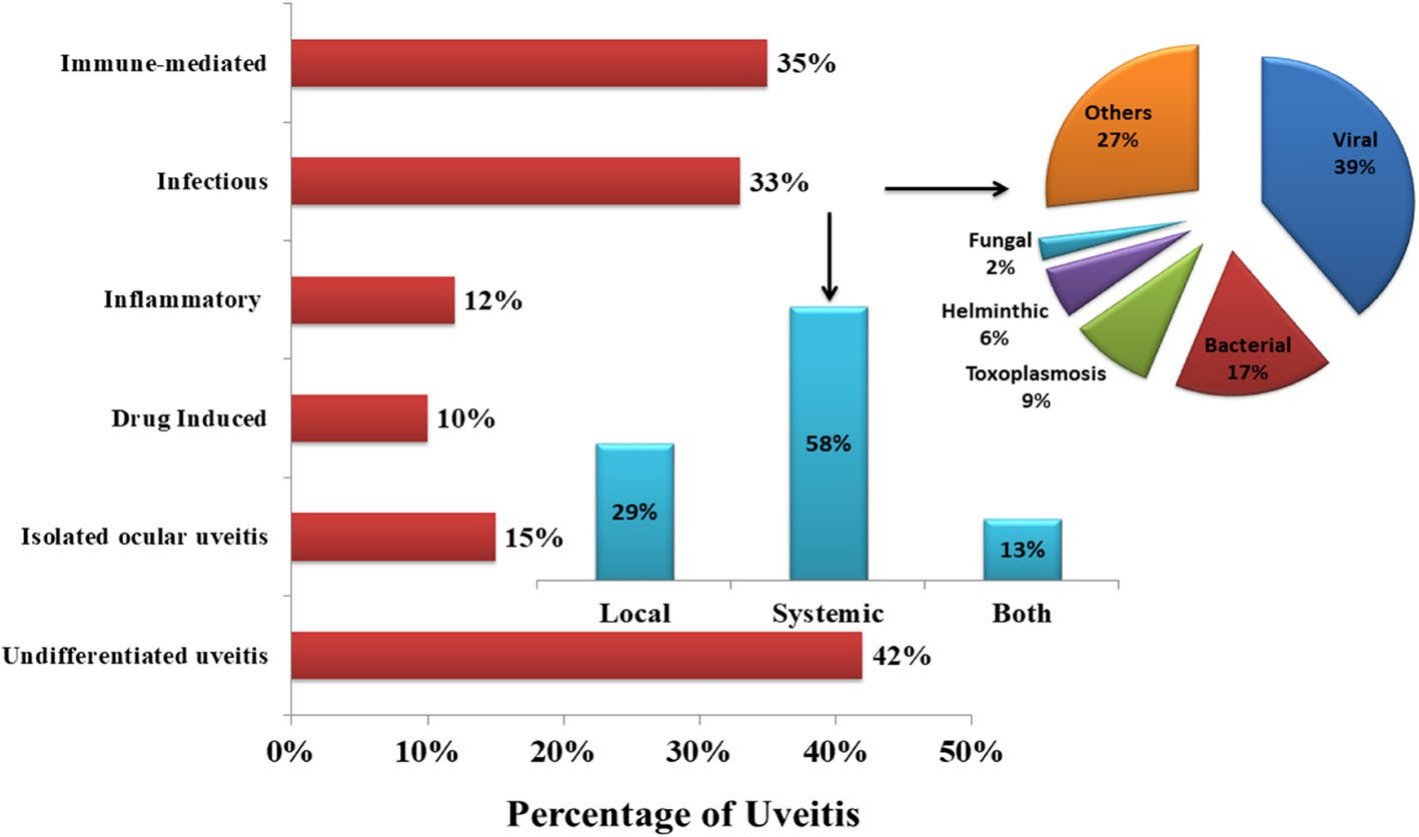
Percentage of reported etiologies for uveitis and further division of infectious uveitis in percentages (Note: Estimates vary widely and should be interpreted with caution due to differences in study populations and methodologies)

More than 60 causes of uveitis have been reported globally and can be classified into six major groups (Fig. 5). The causal epidemiology may vary depending on various factors such as ethnic and genetic factors (sarcoidosis and HLA-B27), disease definition (e.g., sarcoidosis), environmental factors (e.g., tuberculosis), the certain ophthalmic entity’s inclusion in the idiopathic uveitis patients (e.g., pars planitis), patient’s recruitment method (e.g., tertiary centers) and the investigations of paraclinical examinations (e.g., nuclear imaging) [36, 37]. Due to these factors, significant heterogeneity was observed in the reported literature. Figure 5 presents a comprehensive analysis of reported etiologies for uveitis, describing percentages for each category. Among these, immune-mediated uveitis represents 35% of reported cases, while infectious uveitis accounts for 33% [38, 39]. Uveitis caused by inflammation was comprises 12% of cases, while drug-induced uveitis and isolated ocular uveitis contributing 10% and 15% respectively [9, 22]. Undifferentiated uveitis (masquerade syndromes) constitutes the largest proportion at 42% [8]. The major causes of uveitis are found to be infectious, immune-mediated, or some malignancies. The involved association between the human immune system and several infectious agents, including viruses, bacteria, fungi, and parasites, highlights the narrative of uveitis etiology. However, lacked exact values, with estimates based on available information.

Further analysis of infectious uveitis reveals that infections in uveitis can be more confined to the eye region (ocular toxoplasmosis) or with more generalized infections (syphilis, tuberculosis, or Lyme disease) or systemic infections. The summarized data of previously reported worldwide studies also indicated that uveitis is more commonly associated with systemic infections (58%) than local or non-systemic infections (29%) [40–43]. But, further research is needed to confirm these estimates. The predominance of systemic infections underscores the importance of considering broader health implications when evaluating uveitic patients.

Specific types of infectious uveitis are also explained, Fig. 5 shows that viral agents (39%), followed by bacteria (17%) and other infectious agents (27%), are the major causes of infections worldwide, as reported in previous studies [40, 44, 45]. Fungal and helminthic infections contribute 2% and 6% of cases, respectively, while toxoplasmosis accounts for 9% [46–49]. However, these estimates vary widely and should be interpreted with caution due to differences in study populations and methodologies. This detailed analysis provides valuable insights into the distribution of uveitis etiologies, emphasizing the significant proportion of infectious causes and the diversity within this category. Understanding the relative frequencies of different infectious agents can aid clinicians in prioritizing diagnostic tests and selecting appropriate antimicrobial therapies.

### Molecular mechanism of infectious uveitis

#### Protein-protein interaction (PPI) network analysis

**Fig. 6:**
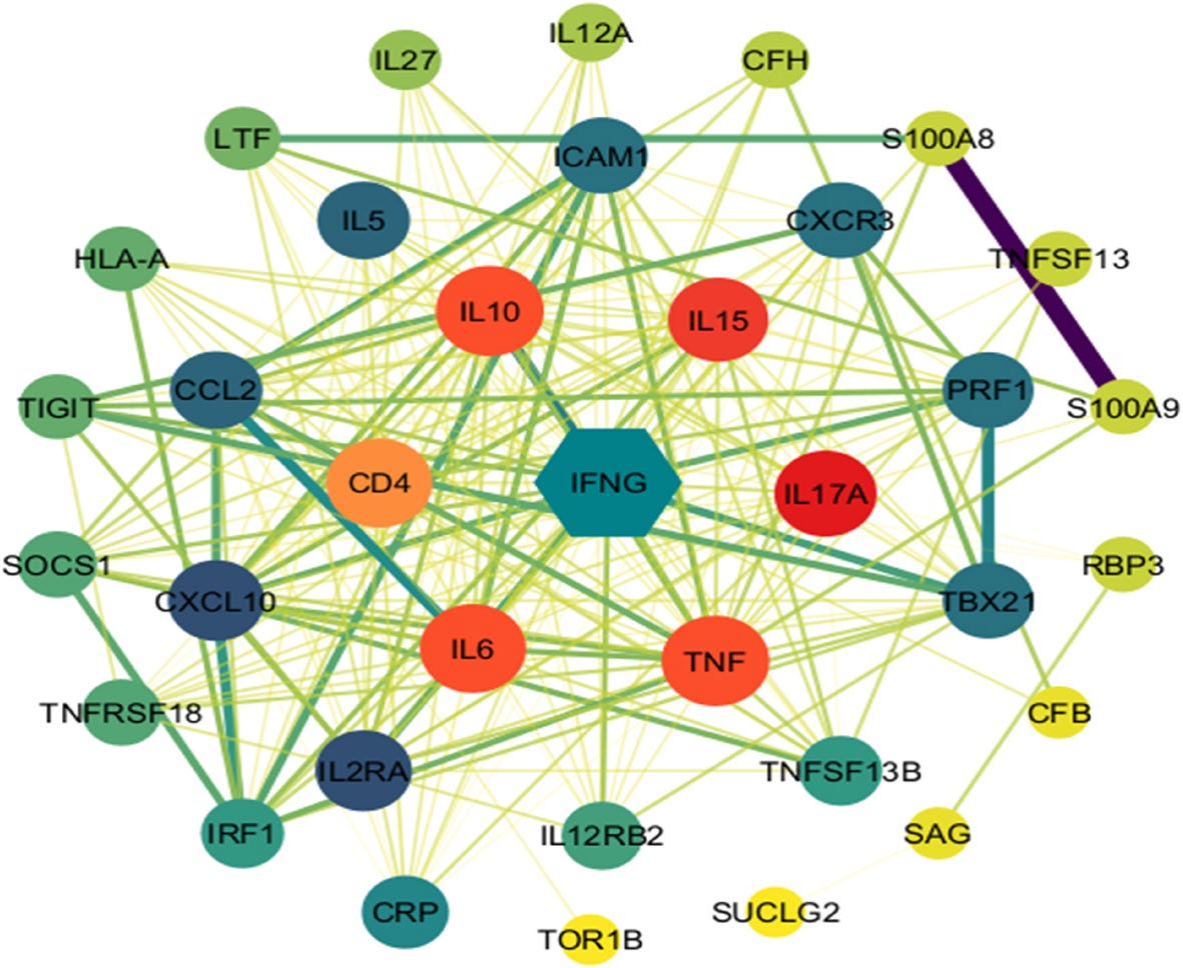
Protein-protein interaction (PPI) network associated with infectious uveitis

The PPI network for infectious uveitis, generated using the STRING database, highlights the centrality of key proteins in the disease’s molecular landscape (Fig. 6). IFNG (Interferon Gamma) emerges as the central node (hub protein), indicating its key role in initiating the immune response. It is known for activating macrophages and promoting antigen production, which are crucial in combating ocular infections [50]. Other highly connected nodes, such as IL6, TNF, IL10, IL17A, and IL15, suggest these cytokines are critical in the inflammatory pathways associated with infectious uveitis. The presence of CD4 in the network points to the involvement of T-helper cells, which play a significant role in adaptive immunity [51]. Inhibitors targeting these cytokines, such as anti-TNF therapies, could be explored for their efficacy in treating infectious uveitis. The centrality and connectivity of certain proteins suggest they could serve as biomarkers for disease activity or therapeutic response. For instance, elevated levels of IFNG, IL6, or TNF in ocular fluids might indicate active inflammation and guide treatment decisions.

#### KEGG pathway and Gene Ontology (GO) enrichment analysis

**Fig. 7:**
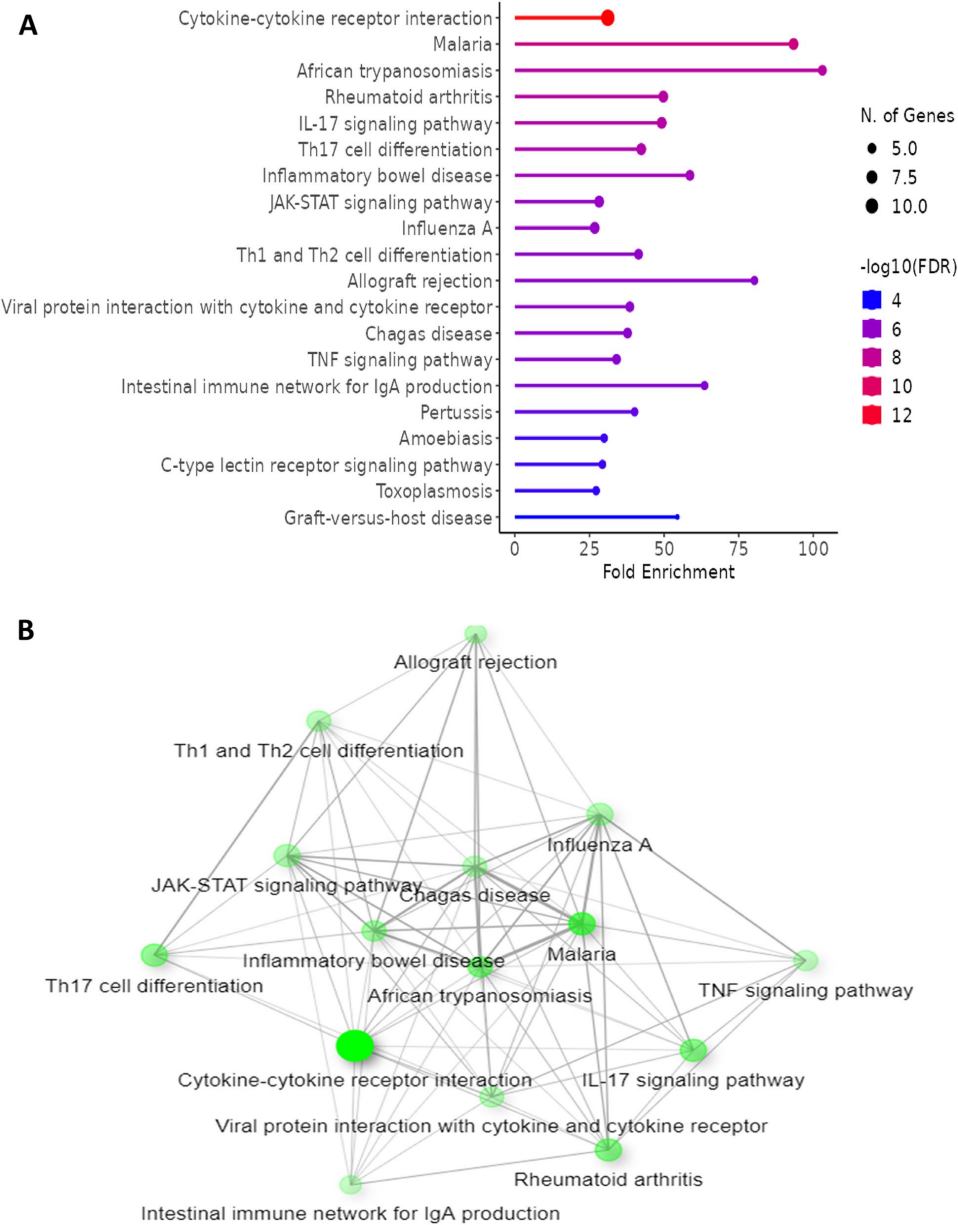
(**A**) KEGG pathway (**B**) KEGG string analysis associated with infectious uveitis

The KEGG pathway analysis identifies several pathways significantly enriched related to infectious uveitis including cytokine-cytokine receptor interaction which highlights the extensive network of cytokine signaling involved in mediating the immune response as indicated in Fig. 7A. JAK-STAT signaling pathway also found known for its role in transmitting signals from cytokine receptors to the nucleus, leading to gene expression changes that cause inflammation [52]. Additionally, modulating the JAK-STAT pathway might offer a therapeutic possibility given its significant role in cytokine signaling. Moreover, Th17 cell differentiation and Th1/Th2 cell differentiation pathways also obtained which are crucial for the polarization of T-helper cells, which can influence the type and severity of the immune response in uveitis [53]. The pathway sting visualization indicates significant cross-talk between pathways such as inflammatory bowel disease, TNF signaling pathway, and IL-17 signaling pathway (Fig. 7B). This interconnectivity suggests that similar molecular mechanisms might be at play in infectious uveitis and other inflammatory diseases. The overlap of enriched pathways with those involved in other inflammatory diseases suggests that therapeutic strategies successful in conditions like inflammatory bowel disease might be modulated for infectious uveitis. The shared molecular mechanisms also provide a framework for understanding the broader immune dysregulation in uveitis.

**Fig. 8:**
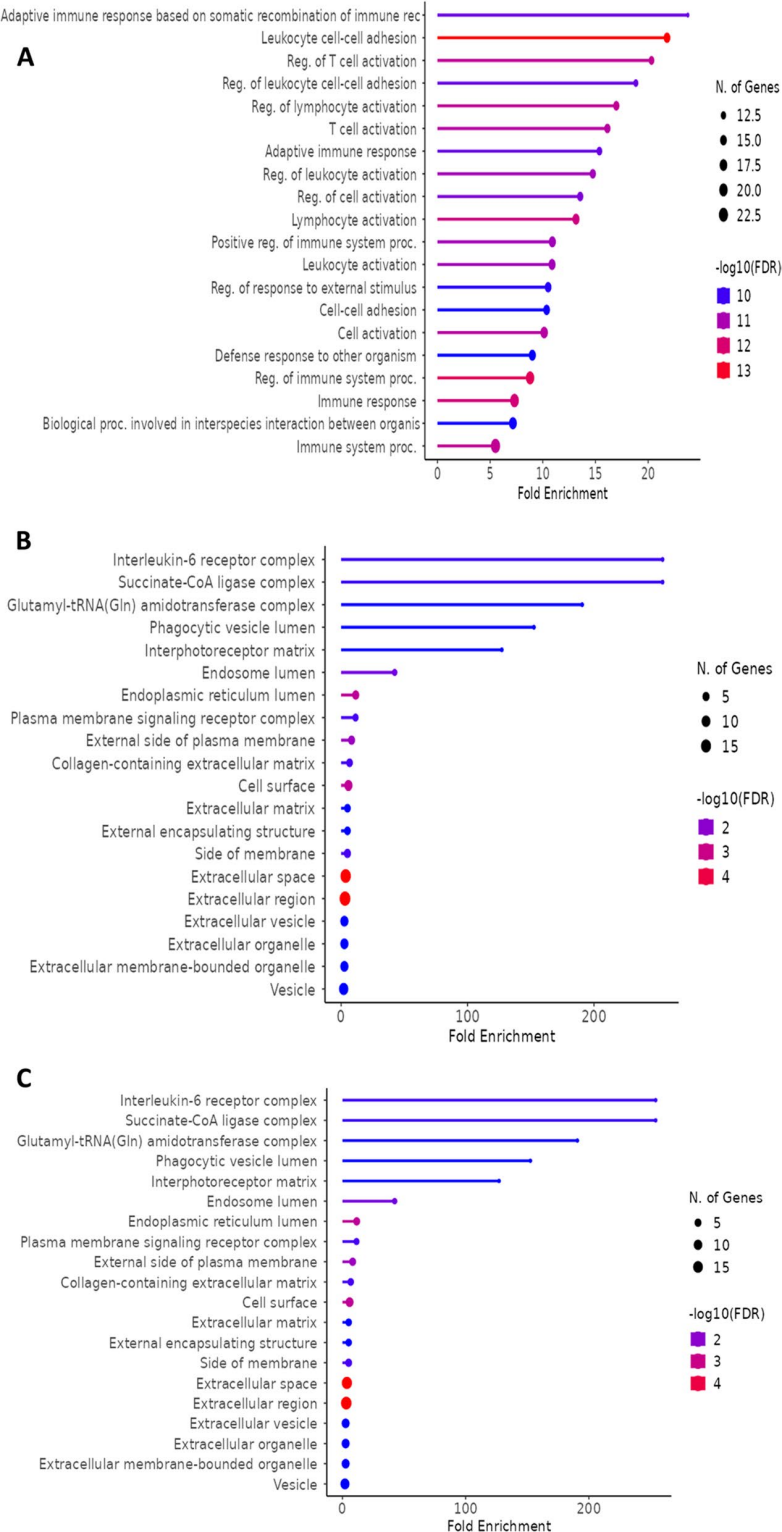
(**A**) Biological enrichment processes (**B**) cellular enrichment processes (**C**) molecular enrichment processes associated with infectious uveitis

GO enrichment analysis for biological processes reveals significant over-representation of immune response-related terms. The top enriched biological processes include: adaptive immune response, regulation of T cell activation and leukocyte cell-cell adhesion (Fig. 8). This indicates the activation of specific immune mechanisms targeting pathogens in the eye. These processes are crucial for the migration and activation of immune cells within ocular tissues [54]. The cellular component enrichment analysis further supports these findings by highlighting the involvement of components such as: Interleukin-6 receptor complex, phagocytic vesicle lumen and endosome lumen (Fig. 8B). It is suggesting a key role for IL-6 signaling in the pathophysiology of infectious uveitis alongside active phagocytosis and antigen processing within immune cells in the eye [55]. Figure 8C showing the molecular component enrichment analysis indicates Toll-like receptor 4, icosatetraaenoic acid binding, and arachidonic acid binding reflecting a significant inflammatory modulation associated with infectious uveitis.

#### Hypothetical pathogenesis of infectious uveitis

**Fig. 9:**
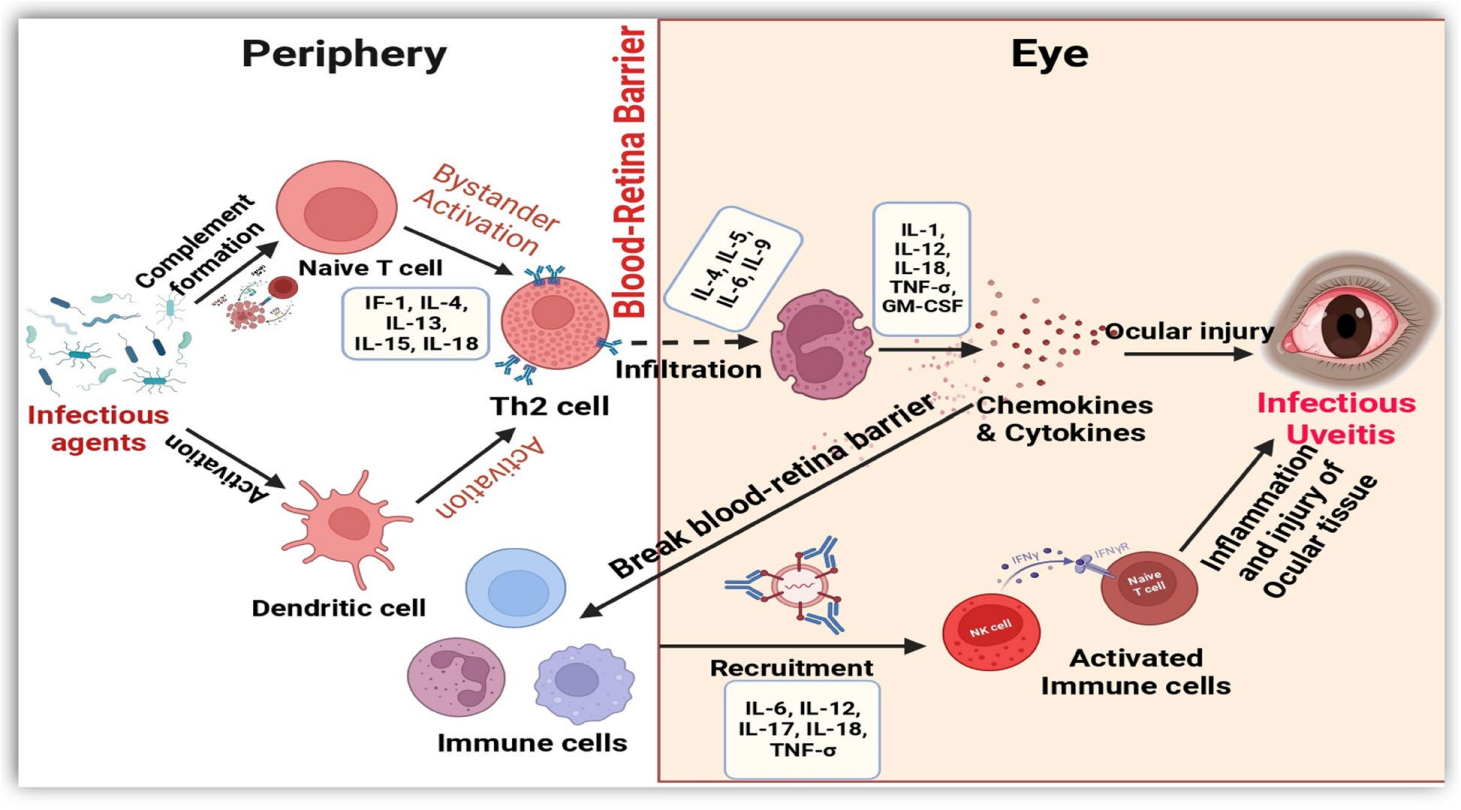
Hypothetical molecular mechanism of infectious uveitis Created in BioRender. Asghar, M. (2024) https://BioRender.com/t53x034

Different viral, bacterial, and parasitic infections have recently been linked with anterior and posterior uveitis and other common ocular manifestations. After successful PPI network, KEGG pathway and GO enrichment analysis associated with infectious uveitis, the complex molecular mechanism of infectious uveitis is also hypothesized in Fig. 9 (Created by BioRender.com), explaining the complex interplay between invading infectious agents and the defense mechanisms of the ocular microenvironment. These infections might trigger inflammatory responses, which have been demonstrated to initiate and exacerbate uveitis. Notably, the immune response, particularly the activation of T-helper type-2 cells, could plays a pivotal role in ocular damage and potential visual loss. Cytokines and chemokines may further propagate the inflammatory signals, coordinating the signaling of various immune cells to the site of infection [56]. The balance between anti-inflammatory mediators and pro-inflammatory signals could influences the severity and duration of infectious uveitis [10]. Moreover, gene expression alterations at the molecular level may also emerged as important contributors to the pathogenesis of infectious uveitis, necessitating further exploration at the genetic level.

**Fig. 10:**
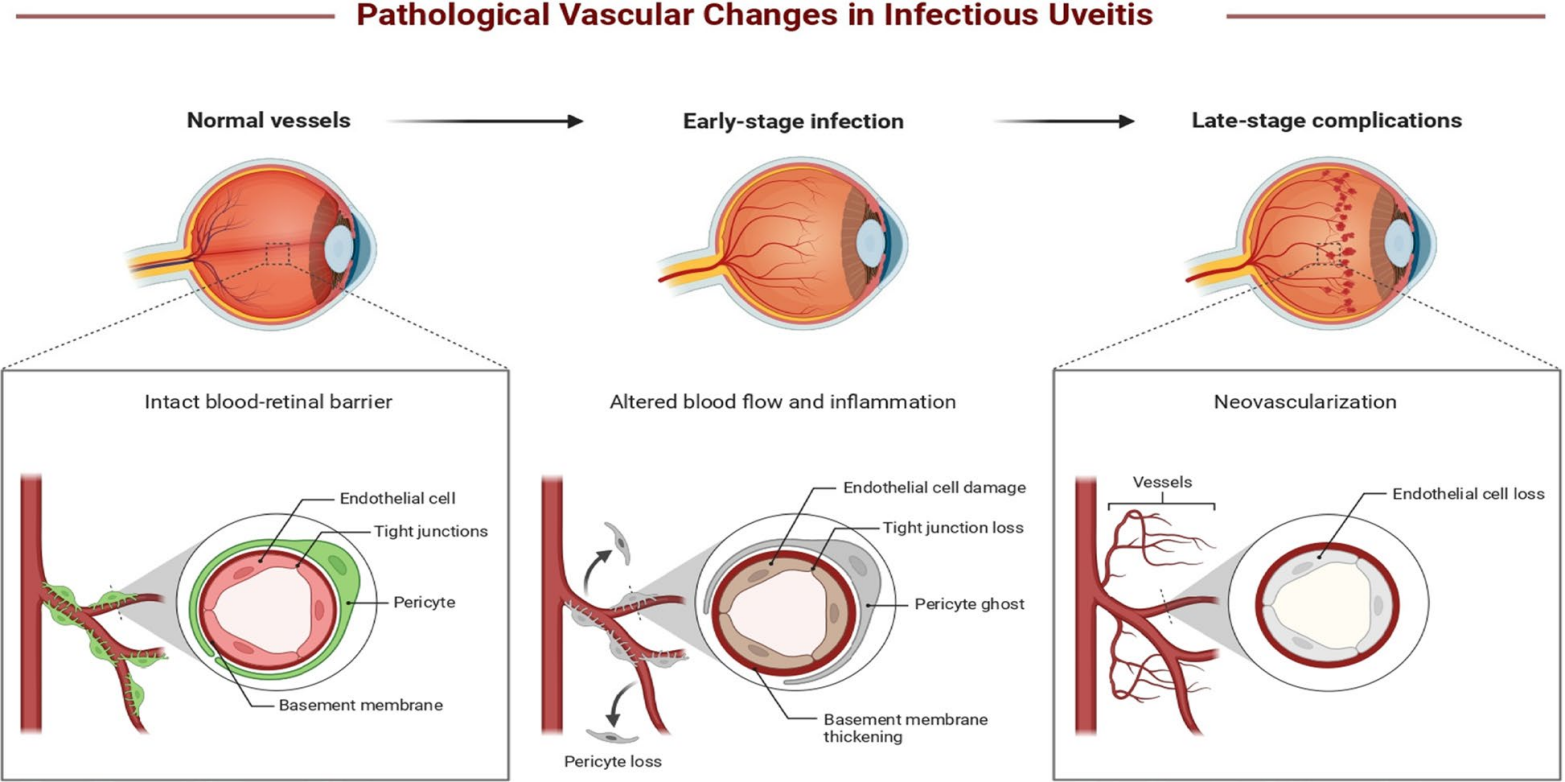
Hypothetical pathological vascular changes in infectious uveitis Created in BioRender. Asghar, M. (2024) https://BioRender.com/t02c088

 Furthermore, pathological vascular changes characteristic of infectious uveitis are predicted in three stages: normal vessels, early-stage infection, and late-stage complications (Fig. 10). The uveal region serves as a focal point for these inflammatory responses, with immune cells in this region and surrounding tissues actively engaging in the inflammatory cascade. It has been reported that infectious uveitis often spreads hematogenously from one part of the body to another, particularly affecting the vascular uvea, often accompanied by breaches in the blood-eye barrier [10, 57]. In infectious uveitis, normal blood vessels with an intact blood-retinal barrier, composed of endothelial cells, tight junctions, pericytes, and a basement membrane, might undergo significant pathological changes. Early-stage infection cloud leads to endothelial cell damage, loss of tight junctions, pericyte loss, and basement membrane thickening, resulting in increased vascular permeability and inflammation. In late-stage complications, these changes may resulted in the loss of endothelial cells and neovascularization, where abnormal and leaky new blood vessels form, further exacerbating tissue damage and inflammation.

Understanding the mechanism of ocular involvement in systemic infectious diseases is crucial in comprehensively elucidating the pathogenesis of infectious uveitis. Virulence factors of pathogens, host susceptibility, and environmental triggers also contribute to the complex pathogenesis of infectious uveitis. Further research focusing on elucidating these molecular and cellular pathways will be invaluable in developing targeted therapeutic strategies to mitigate the detrimental effects of infectious uveitis and improve patient outcomes.

### Clinical manifestations of infectious uveitis

The clinical manifestations of infectious uveitis show a broad spectrum of ocular disorders, varying in severity and presentation depending on the type and nature of the causative pathogen. Infectious uveitis can evoke a range of inflammatory symptoms from mild discomfort to severe, vision-threatening complications. Anterior uveitis, characterized by inflammation of the iris and ciliary body, typically occurs with photophobia, ocular pain, and blurred vision [58]. Conversely, posterior uveitis, involving the retina and choroid, tends to produce more severe symptoms, including visual field defects, floaters, and, in severe cases, permanent blindness. Intermediate uveitis, affecting the peripheral retina and vitreous, may manifest with symptoms such as peripheral vision disturbances and vitreous floaters [59, 60].

Moreover, the clinical manifestations of infectious uveitis can extend beyond ocular symptoms, often presenting with systemic signs and symptoms indicative of the underlying infection. For instance, viral uveitis may be associated with concurrent viral illnesses, such as influenza or herpes simplex virus infection, presenting symptoms such as fever, malaise, and lymphadenopathy [61]. Similarly, bacterial uveitis may manifest with signs of systemic infection, including fever and elevated inflammatory markers [62]. Parasitic uveitis, such as toxoplasmosis, may present with systemic symptoms depending on the extent of the infection [63]. Furthermore, the clinical course and severity of infectious uveitis can vary significantly depending on various factors, including the virulence of the pathogen, host immune response, and timely intervention. Some cases of infectious uveitis may present as acute, self-limiting episodes, while others may progress to chronic inflammation with recurrent flare-ups, leading to irreversible ocular damage and vision loss if left untreated [38]. Understanding the diverse clinical manifestations and natural history of infectious uveitis is crucial for guiding treatment decisions and optimizing patient outcomes.

### Diagnosis and management

The clinical diagnosis and treatment of infectious-associated uveitis pose significant challenges, demanding a comprehensive approach integrating evaluation, specific laboratory investigations, and therapeutic strategies. Differential diagnosis initiates with a detailed patient history encompassing recent infections, systemic illnesses, and other relevant factors linked to specific infections. Clinical examinations, such as fundus fluorescein angiography (FFA) and optical coherence tomography (OCT), are invaluable tools for assessing the nature and severity of uveal inflammation. Confirmation of diagnosis often involves laboratory testing, including serological assays, PCR analyses, and culture techniques aimed at identifying specific infectious agents [38, 64]. Table 1 provides an overview of the investigation processes typically employed in clinical settings for specific types of uveitis associated with common infectious agents. Furthermore, discussion surrounding the diagnosis of infectious uveitis warrants an exploration of viral and non-viral etiologies. Understanding the distinct clinical manifestations, diagnostic techniques, and therapeutic approaches tailored to each infectious agent is paramount for effective management. Viral uveitis, characterized by diverse ocular manifestations, necessitates specific diagnostic modalities such as PCR analysis for viral DNA or antigen detection. Non-viral infectious uveitis, attributed to bacteria, fungi, or parasites, requires careful consideration of serological testing, culture techniques, and imaging modalities to identify the causative pathogen accurately. Emerging technologies, such as multiplex PCR assays and next-generation sequencing, promise to enhance infectious uveitis diagnostic accuracy and efficiency.

**Table 1:** List of investigations for confirmed diagnosis of specific type of infectious uveitis

Type of infectious uveitis	Investigations
Dengue associated uveitis	Detection of dengue IgM antibodies, Fluorescein angiographic examinations, OCT scan
Rift valley fever associated uveitis	Detection of IgM antibodies, Estimate the rising titer of IgG antibody, PCR technique, Fluorescence angiography
West Nile infection associated uveitis	Detection of IgM antibody in infected blood serum or in CSF, OCT scan
Chikungunya associated uveitis	Virus can detected in blister fluid by PCR, Viral genome in their CSF sample, IgM antibodies for Chikungunya, FFA, OCT scan
Zika virus associated uveitis	RT-PCR technique, CT scan, IgM antibody can be detected in CSF
HIV associated uveitis	Count of CD4+, FFA, Detection of HIV related antibodies
Covid-19	RT-PCR, OCT, FFA
Ebola virus infection associated uveitis	RT-PCR, Viral culture technique, OCT scan
Epstein-Barr virus infection associated uveitis	PCR, Histopathological examination of the ocular specimen, Detection of IgM antibodies, Viral capsid antigen (VCA) IgG and IgM can also be detected
Syphilitic uveitis	Rapid plasma regain (RPR), Venereal disease research laboratory (VDRL) test, Microhemagglutinin assay, Fluorescent treponemal, Antibody absorption test
Ocular tuberculosis	Isolation of Mycobacterium TB from ocular region, PCR testing also perform on ocular fluid, Tuberculin skin sensitivity test, Radiological findings, Gamma interferon release assay

The management of infectious-associated uveitis demands a particular and targeted therapeutic approach. Tailored antimicrobial agents, including antiviral, antibiotic, or antifungal medications, are selected based on the identified infectious agents to combat the causative pathogen directly. Additionally, anti-inflammatory agents are crucial in minimizing inflammation and preventing complications related to ocular manifestations.

## Conclusion

This comprehensive review highlights the complex relationship between uveitis and infections, demonstrating the varied clinical manifestations and molecular mechanisms involved. The analysis of the PPI networks and enriched pathways provides crucial insights into potential therapeutic targets, emphasizing the role of cytokines such as IFNG, IL6, and TNF. However, our findings offer significant insights into infectious uveitis; the conclusions remain constrained by the current body of evidence, particularly regarding the variability in diagnostic capabilities and treatment access across different regions. As the understanding of infectious uveitis evolves, there is a need for further epidemiological and molecular studies to validate these pathways and explore new therapeutic interventions, especially those tailored to viral uveitis.

## Future perspective

Future research in the field of infectious uveitis is composed to investigate deeper into the interplay between infectious pathogens and the ocular immune system. The surge in research output, particularly in infectious uveitis, underscores the evolving landscape of this field and the need to address emerging challenges. With the escalating prevalence of infectious diseases and antimicrobial resistance, there is a growing emphasis on exploring innovative antimicrobial-targeted therapies. Moreover, investigating the role of the ocular microbiome in the pathogenesis of infectious uveitis and unravelling the complexities of the ocular immune response hold promise for the development of novel therapeutic interventions.

## Limitations

The limitations of this review include variability in study designs, geographical biases, and inconsistent diagnostic criteria across the included studies. Furthermore, the heterogeneity among studies, particularly in pathogen identification and classification, affects the generalizability of our findings. Additionally, publication bias may have influenced the results, as studies from regions with limited diagnostic resources may underreport infectious uveitis cases. Future research should aim for standardized diagnostic criteria and broader geographical representation to provide a more comprehensive understanding of the global epidemiology of infectious uveitis. In addition, The prevalence of infectious uveitis remains unclear, lacked exact values with available data showing variability depending on the population, limited information and methodology used.

## Data Availability

No datasets were generated or analysed during the current study.

## Abbreviations

CMV: Cytomegalovirus
DAVID: Database for annotation, visualization, and integrated discovery
DisGeNET: Disease gene network
FDR: False discovery rate
FFA: Fundus fluorescein angiography
GO: Gene ontology
HLA-B27: Human leukocyte antigen B27
IL6: Interleukin 6
IFNG: Interferon gamma
JAK-STAT: Janus kinase-signal transducer and activator of transcription
KEGG: Kyoto encyclopedia of genes and genomes
OMIM: Online mendelian inheritance in man
PPI: Protein-protein interaction
PCR: Polymerase chain reaction
STRING: Search tool for the retrieval of interacting genes/proteins
TNF: Tumor necrosis factor
TTD: Therapeutic target database
OCT: Optical coherence tomography

## References

[1] Bonnet C, Brézin A (2020) [Uveitis: diagnosis and work-up]. J Fr Ophtalmol 43(2):145–151 31813553 10.1016/j.jfo.2019.03.038

[2] Burkholder BM, Jabs DA (2021) Uveitis for the non-ophthalmologist. BMJ (Clinical Res ed) 372:m4979 10.1136/bmj.m4979 33536186

[3] Smit RL, Baarsma GS (1995) Epidemiology of uveitis. Curr Opin Ophthalmol 6(3):57–61 10150871 10.1097/00055735-199506000-00010

[4] Accorinti M, Okada AA (2019) Epidemiology of Macular Edema in Uveitis 27(2):169–180 10.1080/09273948.2019.1576910 30821631

[5] Jabs DA (2008) Epidemiology of uveitis. Ophthalmic Epidemiol 15(5):283–284 18850463 10.1080/09286580802478724

[6] Shin Y, Kang JM, Lee J, Lee CS, Lee SC, Ahn JG (2021) Epidemiology of pediatric uveitis and associated systemic diseases 19(1):48 10.1186/s12969-021-00516-2 PMC8015176 33794945

[7] Heiligenhaus A, Rothaus K, Pleyer U (2021) [Development of classification criteria for uveitis by the standardization of uveitis nomenclature (SUN) working group]. Der Ophthalmologe: Z Der Deutschen Ophthalmologischen Gesellschaft 118(9):913–918 10.1007/s00347-021-01486-2 PMC8413183 34459962

[8] Jacquot R, Sève P, Jackson TL, Wang T, Duclos A, Stanescu-Segall D (2023) Diagnosis, classification, and assessment of the underlying etiology of uveitis by artificial intelligence: a systematic review. J Clin Med 12(11):3746 37297939 10.3390/jcm12113746 PMC10253270

[9] Zhang Y, Amin S, Lung KI, Seabury S, Rao N, Toy BC (2020) Incidence, prevalence, and risk factors of infectious uveitis and scleritis in the United States: a claims-based analysis. PLoS ONE 15(8):e0237995 32841267 10.1371/journal.pone.0237995 PMC7447056

[10] Kaburaki T, Fukunaga H, Tanaka R, Nakahara H, Kawashima H, Shirahama S, Izawa H, Komae K, Takamoto M, Soga H (2020) Retinal vascular inflammatory and occlusive changes in infectious and non-infectious uveitis. Jpn J Ophthalmol 64:150–159 32016664 10.1007/s10384-020-00717-4

[11] Takeuchi M, Mizuki N, Ohno S (2021) Pathogenesis of non-infectious uveitis elucidated by recent genetic findings. Front Immunol 12:640473 33912164 10.3389/fimmu.2021.640473 PMC8072111

[12] Page MJ, McKenzie JE, Bossuyt PM, Boutron I, Hoffmann TC, Mulrow CD, Shamseer L, Tetzlaff JM, Akl EA, Brennan SE (2021) The PRISMA 2020 statement: an updated guideline for reporting systematic reviews. BMJ 372:n71 33782057 10.1136/bmj.n71 PMC8005924

[13] Xin W, Zi-Yi W, Zheng J-H, Shao L (2021) TCM network pharmacology: a new trend towards combining computational, experimental and clinical approaches. Chin J Nat Med 19(1):1–11 33516447 10.1016/S1875-5364(21)60001-8

[14] Qu S-Y, Li X-Y, Heng X, Qi Y-Y, Ge P-Y, Ni S-j, Yao Z-Y, Guo R, Yang N-Y, Cao Y (2021) Analysis of antidepressant activity of Huang-Lian Jie-Du decoction through network pharmacology and metabolomics. Front Pharmacol 12:619288 33746756 10.3389/fphar.2021.619288 PMC7970346

[15] Huang F, Fu M, Li J, Chen L, Feng K, Huang T, Cai Y-D (2023) Analysis and prediction of protein stability based on interaction network, gene ontology, and KEGG pathway enrichment scores. Biochim et Biophys Acta (BBA)-Proteins Proteom 1871(3):140889 10.1016/j.bbapap.2023.140889 36610583

[16] Zhang Y-H, Zeng T, Chen L, Huang T, Cai Y-D (2021) Determining protein–protein functional associations by functional rules based on gene ontology and KEGG pathway. Biochim et Biophys Acta (BBA)-Proteins Proteom 1869(6):140621 10.1016/j.bbapap.2021.140621 33561576

[17] Gritz DC, Wong IG (2004) Incidence and prevalence of uveitis in Northern California; the Northern California Epidemiology of Uveitis Study. Ophthalmology 111(3):491–500 (discussion 500) 15019324 10.1016/j.ophtha.2003.06.014

[18] Julian L, Balfour G, Forgues R, de Smet M, Suburo A (2023) Uveitis patterns and severity: an epidemiologic study from a Tertiary Care Private Referral Center in Buenos Aires, Argentina. Ocul Immunol Inflamm 31(4):710–716 35404742 10.1080/09273948.2022.2053546

[19] Radosavljevic A, Agarwal M, Chee SP, Zierhut M (2022) Epidemiology of viral induced anterior uveitis. Ocul Immunol Inflamm 30(2):297–309 33617392 10.1080/09273948.2020.1853177

[20] Tsirouki T, Dastiridou A, Symeonidis C, Tounakaki O, Brazitikou I, Kalogeropoulos C, Androudi S (2018) A focus on the epidemiology of uveitis. Ocul Immunol Inflamm 26(1):2–16 27467180 10.1080/09273948.2016.1196713

[21] Schaftenaar E, Meenken C, Baarsma GS, Khosa NS, Luijendijk A, McIntyre JA, Osterhaus AD, Verjans GM, Peters RP (2016) Uveitis is predominantly of infectious origin in a high HIV and TB prevalence setting in rural South Africa. Br J Ophthalmol 100(10):1312–1316 27307174 10.1136/bjophthalmol-2016-308645

[22] García-Aparicio Á, García de Yébenes MJ, Otón T, Muñoz-Fernández S (2021) Prevalence and incidence of uveitis: a systematic review and meta-analysis. Ophthalmic Epidemiol 28(6):461–468 33557663 10.1080/09286586.2021.1882506

[23] Abaño JM, Galvante PR, Siopongco P, Dans K, Lopez J (2017) Review of epidemiology of uveitis in Asia: pattern of uveitis in a tertiary hospital in the Philippines. Ocul Immunol Inflamm 25(sup1):S75–S80 29083984 10.1080/09273948.2017.1335755

[24] Hao T, Yang L, Li B, Chen X, Li D, Liu X (2021) Epidemiology of 2000 Chinese uveitis patients from Northeast China. Br J Ophthalmol 105(3):317–321 32424058 10.1136/bjophthalmol-2020-316256

[25] Hwang D-K, Chou Y-J, Pu C-Y, Chou P (2012) Epidemiology of uveitis among the Chinese population in Taiwan: a population-based study. Ophthalmology 119(11):2371–2376 22809756 10.1016/j.ophtha.2012.05.026

[26] Rathinam SR, Krishnadas R, Ramakrishnan R, Thulasiraj RD, Tielsch JM, Katz J, Robin AL, Kempen JH, Group ACESR (2011) Population-based prevalence of uveitis in Southern India. Br J Ophthalmol 95(4):463–467 20693551 10.1136/bjo.2010.182311

[27] Sabhapandit S, Murthy SI, Singh VM, Gaitonde K, Gopal M, Marsonia K, Sajid S, Babu K (2017) Epidemiology and clinical features of uveitis from urban populations in South India. Ocul Immunol Inflamm 25(sup1):S39–S45 27782762 10.1080/09273948.2016.1236971

[28] Agrawal R, Thng ZX, Gupta A, Toy BC, Dick AD, Smith JR, Chee SP, Gupta V, Rao NA (2022) Infectious uveitis: conversations with the experts. Ocular Immunology and Inflammation 31(7):1333–1341 10.1080/09273948.2022.2126862 PMC11249092 36345248

[29] Cunningham ET Jr, Khairallah M, Rathinam SR, Belfort R Jr, Zierhut M (2018) Mosquito-Borne Uveitis. Ocul Immunol Inflamm 26(5):651–653 29969369 10.1080/09273948.2018.1485402

[30] Agarwal A, Aggarwal K, Gupta V (2019) Infectious uveitis: an Asian perspective. Eye 33(1):50–65 30315262 10.1038/s41433-018-0224-y PMC6328604

[31] Rathinam SR, Cunningham ET Jr (2000) Infectious causes of uveitis in the developing world. Int Ophthalmol Clin 40(2):137–152 10791262 10.1097/00004397-200004000-00011

[32] Yang P, Zhang Z, Zhou H, Li B, Huang X, Gao Y, Zhu L, Ren Y, Klooster J, Kijlstra A (2005) Clinical patterns and characteristics of uveitis in a tertiary center for uveitis in China. Curr Eye Res 30(11):943–948 16282128 10.1080/02713680500263606

[33] Tabbara KF (2000) Infectious uveitis: a review. Arch De La Sociedad Esp De Oftalmologia 75(4):215–259 11151155

[34] Fox AR, Chew EY, Meyerle C, Vitale S, Ferris FL, Nussenblatt RB, Sen HN (2017) Age-related macular degeneration in patients with uveitis. Br J Ophthalmol 101(3):342–347 27154918 10.1136/bjophthalmol-2016-308587 PMC5589515

[35] Yeung IY, Popp NA, Chan CC (2015) The role of sex in uveitis and ocular inflammation. Int Ophthalmol Clin 55(3):111–131 26035764 10.1097/IIO.0000000000000072 PMC4501391

[36] Kunimi K, Usui Y, Tsubota K, Mitsuhashi R, Umazume A, Kezuka T, Sakai J, Goto H (2021) Changes in etiology of Uveitis in a single Center in Japan. Ocul Immunol Inflamm 29(5):976–981 32068467 10.1080/09273948.2019.1709649

[37] Lee JH, Agarwal A, Mahendradas P, Lee CS, Gupta V, Pavesio CE, Agrawal R (2017) Viral posterior uveitis. Surv Ophthalmol 62(4):404–445 28012878 10.1016/j.survophthal.2016.12.008 PMC5654632

[38] Lin P (2015) Infectious uveitis. Curr Ophthalmol Rep 3:170–183 26618074 10.1007/s40135-015-0076-6 PMC4659396

[39] Odaghi B, Cassoux N, Wechsler B, Hannouche D, Fardeau C, Papo T, Du Le Thi H, Piette JC, Lehoang P (2001) Chronic severe uveitis: etiology and visual outcome in 927 patients from a single center. Medicine 80(4):263–270 11470987 10.1097/00005792-200107000-00005

[40] Yang M, Kamoi K, Zong Y, Zhang J, Ohno-Matsui K (2023) Human immunodeficiency virus and uveitis. Viruses 5;15(2):444 10.3390/v15020444 PMC9962278 36851658

[41] Paez-Escamilla M, Caplash S, Kalra G, Odden J, Price D, Marroquin OC, Koscumb S, Commiskey P, Indermill C, Finkelstein J et al (2023) Challenges in posterior uveitis-tips and tricks for the retina specialist. J Ophthalmic Inflamm Infect 13(1):35 37589912 10.1186/s12348-023-00342-5 PMC10435440

[42] Yildiz Balci S, Turan-Vural E, Turkyilmaz O, Esen F, Aksaray S (2020) Complete blood count parameters and neutrophil-to-lymphocyte ratio values as markers for differentiation between systemic infectious and non-infectious uveitis. Int Ophthalmol 40:3033–3041 32617803 10.1007/s10792-020-01487-1

[43] Yang P, Zhong Z, Du L, Li F, Chen Z, Zhu Y, Zhang W, Huang F, Ye X, Su G (2021) Prevalence and clinical features of systemic diseases in Chinese patients with uveitis. Br J Ophthalmol 105(1):75–82 32188681 10.1136/bjophthalmol-2020-315960

[44] Abroug N, Khairallah M, Zina S, Ksiaa I, Amor HB, Attia S, Jelliti B, Khochtali S, Khairallah M (2021) Ocular manifestations of emerging Arthropod-Borne Infectious diseases. J Curr Ophthalmol 33(3):227–235 34765808 10.4103/joco.joco_134_21 PMC8579803

[45] Goh EJH, Putera I (2023) Ocular Toxoplasmosis 31(7):1342–1361 10.1080/09273948.2022.2117705 36095008

[46] Maruyama K (2019) Current standardized therapeutic approach for uveitis in Japan. Immunological Med 42(3):124–134 10.1080/25785826.2019.1678961 31645201

[47] Takkar B, Venkatesh P (2018) Patterns of uveitis in children at the apex institute for eye care in India: analysis and review of literature. 38(5):2061–2068 10.1007/s10792-017-0700-6 28861733

[48] Khochtali S, Gargouri S, Abroug N, Ksiaa I, Attia S, Sellami D, Feki J, Khairallah M (2015) The spectrum of presumed tubercular uveitis in Tunisia, North Africa. Int Ophthalmol 35(5):663–671 25192914 10.1007/s10792-014-9992-y

[49] Sauer A, de la Torre A, Gomez-Marin J, Bourcier T, Garweg J, Speeg-Schatz C, Candolfi E (2011) Prevention of retinochoroiditis in congenital toxoplasmosis: Europe versus South America. Pediatr Infect Dis J 30(7):601–603 21343838 10.1097/INF.0b013e3182129e70

[50] Ding H, Wang G, Yu Z, Sun H, Wang L (2022) Role of interferon-gamma (IFN-γ) and IFN-γ receptor 1/2 (IFNγR1/2) in regulation of immunity, infection, and cancer development: IFN-γ-dependent or independent pathway. Biomed Pharmacother 155:113683 36095965 10.1016/j.biopha.2022.113683

[51] Zhu X, Zhu J (2020) CD4 T helper cell subsets and related human immunological disorders. Int J Mol Sci 21(21):8011 33126494 10.3390/ijms21218011 PMC7663252

[52] Hu X, Li J, Fu M, Zhao X, Wang W (2021) The JAK/STAT signaling pathway: from bench to clinic. Signal Transduct Target Therapy 6(1):402 10.1038/s41392-021-00791-1 PMC8617206 34824210

[53] Guo K, Zhang X (2021) Cytokines that modulate the differentiation of Th17 cells in autoimmune uveitis. J Immunol Res 2021(1):6693542 33816637 10.1155/2021/6693542 PMC7990547

[54] Mrugacz M, Bryl A, Falkowski M, Zorena K (2021) Integrins: an important link between angiogenesis, inflammation and eye diseases. Cells 10(7):1703 34359873 10.3390/cells10071703 PMC8305893

[55] Egwuagu CE, Alhakeem SA, Mbanefo EC (2021) Uveitis: molecular pathogenesis and emerging therapies. Front Immunol 12:623725 33995347 10.3389/fimmu.2021.623725 PMC8119754

[56] Xu Q, Zhang J, Qin T, Bao J, Dong H, Zhou X, Hou S, Mao L (2021) The role of the inflammasomes in the pathogenesis of uveitis. Exp Eye Res 208:108618 33989670 10.1016/j.exer.2021.108618

[57] Shome A, Mugisho OO, Niederer RL, Rupenthal ID (2021) Blocking the inflammasome: a novel approach to treat uveitis. Drug Discovery Today 26(12):2839–2857 34229084 10.1016/j.drudis.2021.06.017

[58] Sobrin L, Pistilli M, Dreger K, Kothari S, Khachatryan N, Artornsombudh P, Pujari SS, Foster CS, Jabs DA, Nussenblatt RB (2020) Factors predictive of remission of chronic anterior uveitis. Ophthalmology 127(6):826–834 31932091 10.1016/j.ophtha.2019.11.020 PMC7246152

[59] Pichi F, Invernizzi A, Tucker WR, Munk MR (2020) Optical coherence tomography diagnostic signs in posterior uveitis. Prog Retin Eye Res 75:100797 31513851 10.1016/j.preteyeres.2019.100797

[60] Yang P, Yang P (2021) Posterior uveitis. In: Atlas of uveitis: diagnosis and treatment 195–201

[61] Gozzi F, Gentile P, De Simone L, Bolletta E, Alessandrello F, Belloni L, Bonacini M, Croci S, Zerbini A, Cimino L (2022) Viral anterior uveitis. Saudi J Ophthalmol 36(4):356–364 36618575 10.4103/sjopt.sjopt_80_22 PMC9811927

[62] Wildner G, Bansal R, Ayyadurai N, Thurau S, Basu S (2023) Pathogenesis of bacterial Uveitis. Ocul Immunol Inflamm 31(7):1396–1404 36622856 10.1080/09273948.2022.2155842

[63] Gilger BC, Roxane D, Cornelia D (2022) Diseases of the uvea, uveitis, and recurrent uveitis. Equine ophthalmology 441–498

[64] Jeroudi A, Yeh S (2014) Diagnostic vitrectomy for infectious uveitis. Int Ophthalmol Clin 54(2):173–197 24613892 10.1097/IIO.0000000000000017 PMC3979536

